# Staff understanding of recovery-orientated mental health practice: a systematic review and narrative synthesis

**DOI:** 10.1186/s13012-015-0275-4

**Published:** 2015-06-10

**Authors:** Clair Le Boutillier, Agnes Chevalier, Vanessa Lawrence, Mary Leamy, Victoria J Bird, Rob Macpherson, Julie Williams, Mike Slade

**Affiliations:** 1 King’s College London, Health Service and Population Research Department, Institute of Psychiatry, Psychology & Neuroscience, De Crespigny Park, London, SE5 8AF UK; 2 2Gether NHS Foundation Trust, Gloucestershire, UK

**Keywords:** Recovery-orientated practice, Staff perspective, System transformation

## Abstract

**Background:**

Mental health policy is for staff to transform their practice towards a recovery orientation. Staff understanding of recovery-orientated practice will influence the implementation of this policy. The aim of this study was to conduct a systematic review and narrative synthesis of empirical studies identifying clinician and manager conceptualisations of recovery-orientated practice.

**Methods:**

A systematic review of empirical primary research was conducted. Data sources were online databases (*n* = 8), journal table of contents (*n* = 5), internet, expert consultation (*n* = 13), reference lists of included studies and references to included studies. Narrative synthesis was used to integrate the findings.

**Results:**

A total of 10,125 studies were screened, 245 full papers were retrieved, and 22 were included (participants, *n* = 1163). The following three conceptualisations of recovery-orientated practice were identified: clinical recovery, personal recovery and service-defined recovery. Service-defined recovery is a new conceptualisation which translates recovery into practice according to the goals and financial needs of the organisation.

**Conclusions:**

Organisational priorities influence staff understanding of recovery support. This influence is leading to the emergence of an additional meaning of recovery. The impact of service-led approaches to operationalising recovery-orientated practice has not been evaluated.

**Trial Registration:**

The protocol for the review was pre-registered (PROSPERO 2013: CRD42013005942).

**Electronic supplementary material:**

The online version of this article (doi:10.1186/s13012-015-0275-4) contains supplementary material, which is available to authorized users.

## Background

A transformation in mental health systems is underway internationally, towards a focus on promoting recovery [1, 2]. Whilst the recovery process for individuals is influenced by more than their contact with mental health care, services will contribute to many people’s recovery experience [3, 4]. The principle of evidence-based health care is now largely accepted as a quality standard of mental health practice, yet a translational gap between knowledge and routine implementation has been cited as a major challenge to innovation in mental health [5, 6]. National programmes for health service transformation are underway, such as Implementing Recovery through Organisational Change (ImROC) in England [7], but conceptual uncertainty and diverse understandings of recovery-orientated practice present a challenge for mental health professionals and services [8, 9]. Staff perspectives are central to the adoption of recovery-orientated practice, and current evidence identifies the lack of a shared understanding of what recovery means in practice as fundamental to successful implementation [10, 11]. The aim of this study was to conduct a systematic review and narrative synthesis of primary research investigating how clinicians and managers understand recovery-orientated practice in mental health services.

## Method

The review question was *How do clinicians and managers understand the concept of recovery as applied to their practice*? The protocol for the review was pre-registered (PROSPERO 2013:CRD42013005942).

### Eligibility criteria

We sought staff conceptualisations of recovery-orientated practice. Where combined stakeholder conceptualisations of recovery-orientated practice were reported, such as clinician and service user, we included studies where staff made up at least 50 % of participants. We only included English-language articles available in printed or downloadable format.

Participant-inclusion criteria were clinicians and managers, defined as staff from any profession (whether paid or voluntary) who provide or manage mental health services, in primary, secondary or tertiary care. Interventions were explicitly described as pro-recovery. Those typically aligned with recovery e.g. person-centred planning, were only included if identified as recovery-orientated practice. Outcomes were expressed knowledge or attitudes about recovery-orientated practice, or self-reported or observed recovery-orientated behaviour. Finally, study design comprised empirical primary research papers that utilised an established quantitative and/or qualitative research methodology (e.g. questionnaire/survey, interviews, focus groups), with a minimum sample size of three participants.

Studies were excluded if they focused on recovery support in specialist mental health services (e.g. substance misuse, eating disorder) or patient-led organisations (e.g. recovery centres, clubhouse).

### Data sources and search strategy

Due to the complexity of the search area, sequential scoping searches were conducted to test and finalise search terms. The initial search strategy was identified following a review of six pre-selected marker papers, chosen based on expert review of the field. These marker papers were chosen to span a range of study designs and professional groups. The sensitivity of the resulting search was tested by assessing whether the references retrieved from the search included the marker papers. Initial search terms were refined and modified to optimise the balance between specificity and sensitivity. For example, specificity was increased by using terms for specific professional groups to define staff, and the concepts of “understanding” and “applied to practice” were combined to increase sensitivity.

The final protocol comprised search terms identified in the title, abstract, keywords and medical subject headings (MeSH). Search terms were based on the following concepts: all mental illness (not diagnosis specific), recovery (including truncated terms covering recovery orientation, recovery promotion, recovery support etc.) staff (all professional groups) understanding, and applied to practice. The search strategy was designed in OVID and is shown in Additional file 1 online data supplement 1. The strategy was modified for EBSCOhost and PROQUEST.

Six data sources were used as follows:

1. Electronic databases searched from inception until 17 November 2013 were the following: PsycINFO, MEDLINE, EMBASE, Cumulative Index of Nursing and Applied Health Literature (CINAHL), British Nursing Index, International Bibliography of Social Science (IBSS), Applied Social Sciences index and Abstracts (ASSiA) and Scopus.
2. The table of contents from inception until 17 November 2013 were hand searched from Psychiatric Rehabilitation Journal, Psychiatric Services, Journal of Psychiatric and Mental Health Nursing, Administration and Policy in Mental Health and Australian e-journal for Advancement of Mental Health.
3. An internet search using Google Scholar (scholar.google.co.uk) was conducted using the search terms “staff”, “mental health” and “recovery” to identify grey literature of publishable quality. The first 100 entries were reviewed on 10 February 2014.
4. Expert consultation involving 13 mental health service users, professionals, academics and researchers.
5. Reference lists of included articles were hand searched for additional papers.
6. Articles citing included studies were searched using Web of Science (wok.mimas.ac.uk).

### Data extraction

Duplicates were removed in Endnote, Version 6. Titles identified in the electronic search were read, to identify those with possible relevance. Abstracts from relevant publications were reviewed, and where they appeared to meet the inclusion criteria, the full publication was obtained and assessed for eligibility. A random 20 % of the abstracts identified in the database search were independently rated by authors CL and AC for eligibility. One protocol deviation was made following retrieval of full text papers, where the decision was made to exclude studies focusing on the attitudes, knowledge or behaviour of students in professional training. Information was received from three authors (e.g. clarity about the sample) before deciding on inclusion.

All full text papers were independently rated by authors CL and AC for inclusion. Reasons for exclusion were recorded on an eligibility checklist, and disagreements were resolved through discussion or by a third rater (author VL).

### Quality assessment

All included studies were qualitative, so quality was assessed using a framework for assessing qualitative research evidence, covering the different stages and processes within qualitative enquiry and the contribution, defensibility, rigour and credibility of the study [12]. Two raters (authors CL and ML) double-rated the quality of all the included studies. A quantitative score was calculated using the quality framework. Each of the 18 items is rated “yes” (allocated 1 point) or “no” (allocated 0 points), giving a maximum quality rating of 18. High quality was defined as a score of 13 or more, with medium quality papers scoring 7 to 12 and low quality papers scoring 6 or less.

### Analysis

Narrative synthesis was used to analyse the data [13], which involves four stages.

- Stage 1: Develop a theory to inform decisions about the review question and what type of studies to review, to contribute to the interpretation of the review’s findings and to assess how widely applicable those findings may be. A theory is generally developed before synthesis begins, with the aim of the synthesis being to test the limits of theory. In this study, the theory was developed and published by us prior to the review [10]. The theory identified factors that help or hinder clinicians and managers to provide recovery support and to address the lack of a shared understanding of what recovery means in practice.
- Stage 2: Develop a preliminary synthesis, i.e. an initial description of findings from included studies. We used the following two approaches: tabulation and thematic analysis. For each included paper, the following data were extracted: country, service setting, staff group, design and staff sample size. Two analysts (authors CL and AC) independently conducted this tabulation and compared coding decisions to maximise reliability. Disagreements were resolved by discussion. The key terms and components of the described conceptualisation of recovery-orientated practice were then extracted for thematic analysis, to identify the themes occurring within the data. The predefined theory was based on a UK sample, so studies conducted in the UK and Europe were used to identify initial categories; then, studies from other countries were grouped and analysed. To identify main categories and sub-categories, relevant extracts from each text were collated and grouped using a line-by-line approach. An initial deductive coding approach was undertaken whereby categories and sub-categories were mapped onto the stage-one-developed theory. Each category included in the deductive framework was defined to assist consistency of coding between analysts (authors CL and AC). Alongside, an inductive open-coding approach was also undertaken to identify new categories. Analysis was undertaken using NVivo QSR qualitative analysis software, Version 9. Themes were coded at the descriptive level with little attempt to infer beyond the surface or explicit meaning of the text.
- Stage 3: Explore relationships in the data, in order to consider differences within and between the data of included studies. Vote counting was conducted to identify relationships within and between characteristics of each study, including a sub-group analysis by country, profession and health care setting. Thematic vote counting was also conducted using codes and a pre-defined framework of recovery-orientated practice [14].
- Stage 4: Assess the robustness of the synthesis, in order to provide an assessment of the strength of the evidence for drawing conclusions and for generalising the findings of the synthesis. This was achieved through the use of critical appraisal and by placing the findings in the context of wider literature.

## Results

**Fig. 1:**
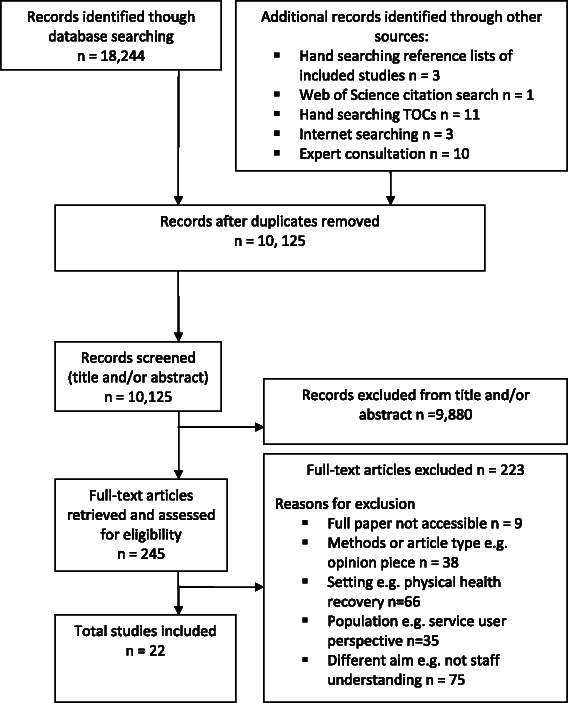
Flow diagram for included studies

The flow diagram is shown in Fig. 1.

### Stage 1: developed theory

Conceptual clarity and staff understanding of recovery-orientated practice is a significant factor influencing the success of implementation, and the theory identified multiple understandings of recovery-orientated practice [10]. Staff struggled to make sense of recovery-orientated practice in the face of conflicting demands, informed by competing priorities of different health system levels. The following three sub-categories outlining the competing priorities were identified: health-process priorities, business priorities and staff-role perception. The health-process-priorities category linked with the concept of clinical recovery and reflected traditional mental health concerns, including a focus on symptomatology and functioning and the evidence-based medicine view of scientific knowledge. The business-priorities category highlighted how financial and organisational priorities influence practice, viewing recovery as a service outcome, with potential trade-offs between quality and quantity. The final category, staff-role perception, captured staff views of their role and individual priorities in supporting recovery, which ranged from a custodial orientation to a recovery-orientated model of care, with a corresponding focus of practice from narrow (primarily symptomatology) to a more holistic emphasis.

### Stage 2: preliminary synthesis and tabulation

All 22 papers included in the review were qualitative or mixed-method studies (incorporating a qualitative component) reporting a staff conceptualisation of recovery-orientated practice. The total number of participants was 1163. Study designs comprised interview (*n* = 10), focus group (*n* = 6), interview and focus group (*n* = 2), participant observation (*n* = 1), Delphi consultation (*n* = 1) and mixed-methods (*n* = 2) study designs. Studies involved nurses (*n* = 3), case managers (*n* = 3), social workers (*n* = 2), psychiatrists (*n* = 2), team leaders (*n* = 1), occupational therapists (*n* = 1), clinical psychologists (*n* = 1), art therapists (*n* = 1) and multidisciplinary samples (*n* = 8). Service settings were in-patient (*n* = 5), community (*n* = 8), both (*n* = 7) or not specified (*n* = 2). Research took place in USA (*n* = 7), Australia (*n* = 4), Canada (*n* = 4), UK (*n* = 3) and Europe-wide (*n* = 1), Hong Kong (*n* = 1) and Thailand (*n* = 1). Included studies are summarised in Additional file 1 online data supplement 2, and the data extraction table characterising each study is shown in Additional file 1 online data supplement 3.

### Thematic analysis

The thematic analysis of the four UK and European papers led to an initial framework with one overarching category, called staff-role perception. Staff conceptualisations of recovery-orientated practice fell into the following three sub-categories: clinical recovery, personal recovery and service-defined recovery. These themes were then developed and extended further using the 18 studies conducted outside Europe. No further categories were identified, suggesting that the developed theory is not specific to the UK context.

### Overarching category: staff-role perception

There are differences in how staff perceive their role in supporting recovery. Nine papers reported conceptual uncertainty, finding that recovery-orientated practice is a “difficult to define” concept [15, 16]. Aston and Coffey (2012) found that *all participants had difficulty in articulating what recovery meant to them and its application to mental health* [15]. It was therefore not surprising that “there is still considerable confusion about what mental health systems and psychiatrists should be achieving in a recovery-oriented system” [17]. Other authors wrote:

There were comments that there is no theoretical base in the recovery approach, it is an approach it is not a model, there is no clear definition of recovery or there are several definitions [18].

Providers expressed support of the philosophical tenets of recovery, but seemed unsure of how to operationalize recovery in a meaningful way. But these women are struggling to keep their head above water, get basic stuff done. Recovery can seem almost like an unattainable goal, that doesn’t have a lot of meaning for poor women [19].

Some staff were confused by their role due to the uncertainty of what recovery means in practice:

Given the multiple models of recovery from mental illness, providers were perplexed by what exactly was expected of them as publicly funded caregivers, as well as of the consumers they serve. Did recovery represent an outcome or a process? [20]

In other words, the rhetoric of “recovery” is being used in services without clear understanding (Tickle 2012), with the suggestion that “many practitioners had jumped on the bandwagon without fully exploring what recovery means for practice” [21].

Other studies found that recovery-orientated practice is not a new concept for staff:

Other participants argued that recovery-oriented reforms within their organizational contexts did not contribute anything new to their practice. They emphasized that that they were already implementing recovery long before it became a politicized concept. They characterized the term recovery as a “buzz word” or “fad” in mental health discourse, and a re-invention of what already existed [22].

“It’s just what clinicians do”, “It just feels like common sense at times” [23].

The need for a shared understanding of recovery-orientated practice was identified as follows:

It is evident, however, that there is more than one understanding of recovery, that these are sometimes idiosyncratic and that accomplishing a form of shared understanding is crucial to achieving mental health service-facilitated recovery [15].

### Sub-category 1: clinical recovery

Clinical recovery was defined as a deficit perspective where mental state is improved or stabilised using medication and risk-management interventions. Clinical recovery was measured by symptom remission, insight gain, absence of relapse and mastery in daily living skills. The focus was on the professional as an expert working within an established health infrastructure, with clinical tasks shaping recovery-orientated practice.

Nurses viewed recovery from schizophrenia as involving symptom stabilization and the restoration of psychosocial functioning. Their views of recovery were characterized by a focus on clinical and functional improvement, such as symptom remission, an ability to carry out daily living activities, and a return to work or study [24].

The power of the psychiatrist in assessing the patient to be relapse-free was noted in the following study-participant quotes:

S2: “I must assess how long a patient can remain relapse-free before I can declare my patient as having recovered.”

J3: “A perfect recovery should imply no relapse.”

J4: “If we cannot guarantee absence of relapse in the next 30 years, how can we say a patient has recovered?”

S5: “We have to assess how long a patient can remain relapse-free before we can define the patient as having recovered, much like the concept of ‘survival rate’ in cancer” [16].

Insight in the patient was linked with recovery orientation:

Sometimes, for your folks who understand, ‘I am not well right now. something is the matter”… recovery makes sense. They have a grasp on their illness and they know they are not feeling well, versus I could be feeling better. For other folks who don’t have insight into why you are in their life at all, recovery doesn’t work [25].

### Sub-category 2: personal recovery

Personal recovery was defined as a holistic approach (spanning physical health care, psychological therapies and stress management) where individuality (including client-centred goals, service-user autonomy and decision-making) takes precedence, and staff and service users work in partnership (through, for example, coaching, supporting hope). Personal recovery was measured by citizenship involvement (including meaningful occupation and social inclusion).

A power shift is involved in client-centred personal recovery support:

Recovery was viewed as individually determined and predicated primarily on what consumers wanted, not on what professionals perceived as the upper limits of what is possible [25].

The most prominent idea that emerged when respondents were asked what the concept of recovery means to them is that of holism. This included social factors such as relationships, psychological issues like self-esteem, and practical matters such as living skills, money, education and work [26].

Autonomy and decision-making are important components of personal recovery support:

“It becomes their choice whether they do these things or not or they can also decide that whatever was initially important isn’t important anymore. That’s up to them. But if they…if it’s still important, then they got to do certain things.”

“In the end, it doesn’t matter what my thoughts are about discharge planning. It’s about what the client wants and is willing to do.” [27].

Supporting hope was a prominent theme:

You have to be able to bring your clients along with you … and have them as invested as much in their recovery as you are. So that is the skill. The most important thing is knowing how to do that, and then holding that vision for them when they can’t…sometimes they can’t envision their recovery [28].

### Sub-category 3: service-defined recovery

Service-defined recovery was defined as a concept owned by the organisation where administrative and financially driven goals shape practice. Service-defined recovery was viewed as a tool to reduce costs and measured by service throughput (including discharge) and service accessibility.

Financial and administrative priorities dictate practice:

Current mental health reimbursement systems do not support recovery. Participants pointed out that federal, state, and local public mental health systems have not framed financial reimbursement systems to reflect recovery-oriented care. Despite the emphasis on recovery in public statements and formal planning documents, public mental health providers are still primarily focused on symptom remission and client stabilization, with limited opportunities to expand the number of reimbursable programs that emphasize community integration and recovery [17].

Recovery orientation can be viewed by staff as something owned by the organisation and therefore supported in order to meet organisational targets:

Recovery was identified by several participants as a Trust ‘initiative’. Despite recognition that the Trust was committed to recovery, there was a lack of clarity about what the Trust meant by recovery, how it related to other initiatives and Trust strategies, and in particular what this meant in terms of the role of services. This led some interviewees to suggest that a recovery approach was being implemented for political reasons, to meet government targets, as a tool for reducing costs, and like previous initiatives, may soon be de-prioritised [18].

Service users can therefore receive the message that recovery support will mean reduced service input:

“Providers expressed frustration with their role to aid women in recovery. Although participants spoke positively about recovery, the implementation of this guiding vision was fraught with difficulties. I have to say that I am really for the idea of recovery, (laughs); I just want to go on record that I am for recovery! But whatever that means for that person, you know. I know so many women that are confused about the whole idea, I try to talk to them about recovery and they ask me ‘does that mean you don’t want to see me?” [19].

The three conceptualisations of recovery-orientated practice are not mutually exclusive, and some staff understand their role in supporting recovery as integrative:

Here, ‘medical’ intervention is equated with involuntary treatment and medication, and deemed to be just as important as ‘recovery’. Thus, Paul attributed successful recovery to a worker’s ability to apply equal value to both dimensions of practice by balancing these competing needs against each other [21].

### Stage 3: exploring the relationships between studies

All 22 studies were included in the vote-counting process. For the category personal recovery, papers were characterised using categories from an existing conceptual framework of recovery-orientated practice [14], as shown in Table 1.

**Table 1:** Vote counting for personal recovery sub-category

Personal recovery category																	
Study ID	Promoting citizenship				Organisational commitment					Supporting personally defined recovery					Working relationship		
	Seeing beyond service user	Service user rights	Social inclusion	Meaningful occupation	Recovery vision	Workplace support structures	Quality improvement	Care pathway	Workforce planning	Individuality	Informed choice	Peer support	Strengths focus	Holistic approach	Partnerships	Inspiring hope	Total
Aston 2012 [15]																	0
Gilburt 2013 [18]			X	X										X	X	X	5
Tickle 2012 [35]				X			X				X						3
Turton 2010 [36]		X	X				X	X		X			X	X		X	8
Felton 2006 [29]											X			X	X		3
Sullivan 2013 [25]											X				X	X	3
Sullivan 2012 [28]											X					X	2
Rice 2009 [19]																	0
Watson 2011 [20]			X									X					2
Rogers 2007 [17]			X														1
Dunlap 2009 [27]		X								X	X		X		X	X	6
Courtney 2013 [21]		X								X					X		3
Vanlith 2009 [37]				X										X		X	3
Cleary 2013 [26]														X			1
Hungerford 2013 [38]																	0
Battersby 2012 [39]			X	X						X	X					X	5
Schwartz 2013 [40]														X			1
Kidd 2014 [41]	X										X				X		3
Piat 2012 [22]														X	X		2
Ng 2008 [16]																	0
Kaewprom 2011 [24]			X					X									2
Cone 2012 [23]			X	X									X	X			4
Total	1	3	7	5	0	0	2	2	0	4	7	1	3	8	7	7	57

Individual studies contained a mean of 2.6 (16 %, range 0 to 8) of the 16 categories of personal recovery. The categories with the most studies were holistic approach and partnerships (eight studies each) followed by social inclusion, informed choice and inspiring hope (seven studies each).

For the clinical recovery and service-defined recovery categories, papers were characterised using lower-order sub-categories developed from the synthesis. Table 2 shows the vote counting for the clinical-recovery category.

**Table 2:** Vote counting for clinical recovery sub-category

Study ID	Clinical recovery									
	Deficit perspective	Medication adherence	Symptom remission	Gaining insight	Absence of relapse	Risk/crisis management	Meet basic survival needs	ADL task mastery	Stabilising or fixing patients	Total
Aston 2012 [15]	X								X	2
Gilburt 2013 [18]	X	X	X			X			X	5
Tickle 2012 [35]						X				1
Turton 2010 [36]		X								1
Felton 2006 [29]		X		X		X		X	X	5
Sullivan 2013 [25]		X		X					X	3
Sullivan 2012 [28]										0
Rice 2009 [19]										0
Watson 2011 [20]	X		X							2
Rogers 2007 [17]			X			X			X	3
Dunlap 2009 [27]	X					X				2
Courtney 2013 [21]	X	X								2
Vanlith 2009 [37]										0
Cleary 2013 [26]		X	X			X				3
Hungerford 2013 [38]										0
Battersby 2012 [39]	X									1
Schwartz 2013 [40]						X			X	2
Kidd 2014 [41]						X				1
Piat 2012 [22]						X	X			2
Ng 2008 [16]		X	X		X			X	X	5
Kaewprom 2011 [24]		X	X		X			X	X	5
Cone 2012 [23]										0
Total	6	8	6	2	2	9	1	3	8	45

Individual studies contained a mean of 2.1 (23 %, range 0 to 5) of the nine sub-categories of clinical recovery. The sub-categories with the most studies were risk/crisis management (nine studies), medication adherence and stabilising or fixing patients (eight studies each).

Table 3 shows the vote counting for the service-defined recovery category.

**Table 3:** Vote counting for Service-defined recovery category

Study ID	Service-defined recovery							
	Owned by the organisation	Administrative/financially driven goals	A tool to reduce costs	Service throughput/moving-on	Discharge	Reducing service accessibility	Setting limits on service provision	Total
Aston 2012 [15]								0
Gilburt 2013 [18]	X			X				2
Tickle 2012 [35]					X			1
Turton 2010 [36]								0
Felton 2006 [29]		X			X	X		3
Sullivan 2013 [25]								0
Sullivan 2012 [28]								0
Rice 2009 [19]					X			1
Watson 2011 [20]		X			X		X	3
Rogers 2007 [17]		X						1
Dunlap 2009 [27]		X			X		X	3
Courtney2013 [21]			X				X	2
Vanlith 2009 [37]								0
Cleary 2013 [26]				X				1
Hungerford 2013 [38]								0
Battersby 2012 [39]								0
Schwartz 2013 [40]								0
Kidd 2014 [41]								0
Piat 2012 [22]	X	X						2
Ng 2008 [16]								0
Kaewprom 2011 [24]								0
Cone 2012 [23]		X						1
Total	2	6	1	2	5	1	3	20

Individual studies contained a mean of 0.9 (13 %, range 0 to 3) of the seven sub-categories of service-defined recovery. The sub-categories with the most studies were administrative/financially driven goals (six studies) and discharge (four studies).

The primary focus of personal recovery was a holistic approach and an emphasis on social inclusion, choice and hope-inspiring partnership working. The primary focus of clinical recovery was risk, medication and clinical management. The primary focus of service-defined recovery was a focus on organisational goals and on discharge. Overall, staff understandings spanned personal, clinical and service-defined recovery with the strongest mapping for clinical recovery (23 %) and the weakest mapping for service-defined recovery (13 %).

Included studies were spread across all three conceptualisations of recovery-orientated practice with no difference in country, setting or professional groups. The characteristics (country, study setting, participant and professional group(s)) of each study (*n* = 22) are detailed in Table 4 alongside vote counting of the three different conceptualisations of recovery-orientated practice.

**Table 4:** Vote counting across three conceptualisations of recovery in practice

#	Study ID	Country	Profession	Setting	Clinical recovery	Personal recovery	Service-defined recovery
1	Aston 2012 [15]	UK	Nurses	In-patient	X		
2	Gilburt 2013 [18]	UK	Team leaders	Across settings	X	X	X
3	Tickle 2012 [35]	UK	Clinical psychologists	Across settings	X	X	X
4	Turton 2010 [36]	Europe	MDT	In-patient	X	X	
5	Felton 2006 [29]	USA	MDT	Community	X	X	X
6	Sullivan 2013 [25]	USA	Case managers	Community	X	X	
7	Sullivan 2012 [28]	USA	Case managers	Community		X	
8	Rice 2009 [19]	USA	Case managers	Community			X
9	Watson 2011 [20]	USA	MDT	Across settings	X	X	X
10	Rogers 2007 [18]	USA	Psychiatrists	Across settings	X	X	X
11	Dunlap 2009 [27]	USA	Social workers	Not known	X	X	X
12	Courtney 2013 [21]	Australia	Social workers	Community	X	X	X
13	Vanlith 2009 [37]	Australia	Art therapists	Community		X	
14	Cleary 2013 [26]	Australia	Nurses	In-patient	X	X	X
15	Hungerford 2013 [38]	Australia	MDT	Not known		X	
16	Battersby 2012 [39]	Canada	MDT	Community	X	X	
17	Schwartz 2013 [40]	Canada	MDT	Community	X	X	
18	Kidd 2014 [41]	Canada	MDT	In-patient	X	X	
19	Piat 2012 [22]	Canada	MDT	Across settings	X	X	X
20	Ng 2008 [16]	Hong Kong	Psychiatrists	Across settings	X		X
21	Kaewprom 2011 [24]	Thailand	Nurses	In-patient	X	X	
22	Cone 2012 [23]	New Zealand	Occupational therapists	Across settings		X	X
Total					17	19	12

Year of publication was not a factor in determining staff understanding indicating that conceptualisations have been evident for a while. For example, the oldest study (published in 2006) mapped all three conceptualisations of recovery as applied to practice [29].

High-quality and low-quality studies did not differ in their profiles and also referred to all three conceptualisations of recovery. Three of the four studies assessed as high quality (scored 13 or 14 out of a possible 18) identified all three conceptualisations in the findings [22, 27, 29]. The study identified as the lowest quality (scored 2 out of a possible 18) also highlighted the three conceptualisations of recovery as applied to practice [17].

### Stage 4: assessing the robustness of the synthesis

To ensure a robust synthesis, methodological rigour and data checking was undertaken at each stage of the data collection and analysis. A random 20 % (*n* = 2,033) of sifted papers were double-rated, with agreement on 1972 (97 %). The 61 papers with discordant ratings were obtained in full, and 2 (3 %) were assessed as eligible for inclusion. All 245 papers retrieved in full were double-rated for inclusion, with 95 % concordance. Data relevant to the research question from all included studies were extracted and tabulated independently by two analysts (CL and AC). Finally, the thematic analysis of the preliminary framework using UK and European studies was completed separately by two analysts.

## Discussion

The aim of the review and narrative synthesis was to obtain conceptual clarity about staff understanding of recovery-orientated practice. A total of 22 studies describing staff conceptualisations of recovery-orientated practice were included. Narrative synthesis identified three staff conceptualisations of recovery-orientated practice such as: clinical recovery, personal recovery and service-defined recovery. The concepts of clinical recovery and personal recovery are well documented [30, 31]. Service-defined recovery extends the meaning of recovery-orientated practice by translating recovery into practice according to the goals and financial needs of the organisation.

Our review found a lack of theoretical clarity about the task of supporting recovery in practice. There is inconsistency in the influence of policy direction, funding decisions and organisational priorities on service delivery. Organisational priorities influence how staff understand recovery-orientated practice, so service-defined recovery will influence the delivery, management and evaluation of recovery-orientated practice. This new understanding of recovery is consistent with concerns raised by people who use mental health services about the misuse of recovery to meet service demands (focusing on reduced financial expenditure rather than quality) which do not align with the priorities of service users [32] and focus on organisational goals rather than their own [33].

### Strengths and limitations

This is the first systematic review and narrative synthesis of staff conceptualisations of recovery-orientated practice. Until now, staff perspectives have been largely absent from the recovery literature. This is consistent with the present review in which only 22 of the 245 papers accessed in full and assessed for eligibility focused on staff understanding. Adopting a transparent systematic review and narrative synthesis methodology addresses some of the criticisms regarding rigour and increases confidence in the findings. The robustness of the review was enhanced by two approaches for validating the framework, namely the double-rating of a proportion of papers to assess eligibility, and double-coding and data extraction of included papers.

A limitation was that the narrative synthesis is a secondary analysis of data that focuses on the interpretations presented by the authors of the original papers and is not based on primary data. Furthermore, the findings represent one interpretation of the data and should be viewed as a heuristic theory of staff perspectives on recovery-orientated practice.

### Practice implications

National mental health policy identifies “personal recovery” as the intended orientation of mental health services [1]. Our review indicates that a shift from the dominant clinical recovery paradigm to personal recovery is underway, but not complete. However, the emergence of a new “service-defined understanding of recovery” has unknown implications for mental health systems. The clear differentiation between different understandings of recovery held by staff provides a framework for assessing the recovery orientation of mental health services and can be used to develop accreditation criteria and fidelity indicators.

Given the absence of nationally endorsed clinical guidelines for recovery-orientated care, it is not surprising that management tools and process indicators (throughput, discharge etc.) have been used by organisations to define recovery [10]. However, this attempt to operationalise recovery through the lens of organisational priorities has been criticised, both by people working in the system [34] and by people who use services [32]. The outcome and resource implications of service-defined recovery are unknown, so cost-effectiveness studies are a priority for future research.

Staff have to balance competing demands in relation to recovery [10]. The increasing emphasis on organisational management of a previously more autonomous work-force in the service of efficiency and budgetary management means that service-defined recovery is increasingly evident. The danger is that a focus on meeting the needs of the organisation may take priority over the provision of client-centred recovery support. It is reasonable to expect that there are trade-offs between the outcomes arising from a focus on clinical, personal and service-defined recovery. For example, maximising treatment adherence (for clinical recovery), choice (for personal recovery) and reducing service use (for service-defined recovery) may not be possible. No stake-holder’s interests are served if incompatible and unmeetable expectations are placed on staff to fully support all three types of recovery.

This clarification of staff understanding of recovery-orientated practice indicates that organisational transformation towards a recovery orientation needs to be as focused on how the mental health system is managed as on the interventions being provided.

## Additional file

Additional file 1: **Online data supplement 1: search strategy. Online data supplement 2: summary of included studies (*****n*** **= 22).** Online data supplement 3: data extraction table (*n* = 22).
