# Supplementary material for: Staff understanding of recovery-orientated mental health practice: a systematic review and narrative synthesis
Source: Implement Sci. 2015 Jun 10;10:87. doi: 10.1186/s13012-015-0275-4 (PMC4464128; doi:10.1186/s13012-015-0275-4)
Supplement: Additional file 1: — Online data supplement 1: search strategy. Online data supplement 2: summary of included studies (n = 22). Online data supplement 3: data extraction table (n = 22). [file 13012_2015_275_MOESM1_ESM.doc]

('mental health' OR 'mental illness' OR 'mental disorder' OR 'mental disease' OR mental adj2 problem$ OR psychol$ adj2 (health or problem$ or disorder$ or illness$) OR psychiatr$ adj2 (health or illness$ or disorder$ or problem$ or disabilit$))

AND

'recover$'

AND

(staff OR worker$ OR “care coordinator$” OR personnel OR employee$ OR clinician$ OR professional$ OR practitioner$ OR provider$ OR leader$ OR manager$ OR physician$ OR psychiatrist$ OR doctor$ OR nurse$ OR "occupational therapist$" OR "social work$" OR psychologi$ OR "peer support$" OR "vocational specialist$" OR volunteer$ OR student$ OR "decision maker$")

AND

(mean$ OR define$ OR comprehen$ OR opinion$ OR view$ OR belief$ OR knowledge$ OR perspective$ OR attitude$ OR discourse$ OR theor$ OR experience$ OR perception$ OR rhetoric OR awareness OR translat$ OR implement$ OR operationali$ OR philosoph$ OR appl$ OR understand$ OR conceptuali$ OR interpret$ OR value$ OR behavio$).

**Online Data supplement 1: Search strategy**

| **#** | **Study ID** | **Country** | **Setting** | **Staff group** | **n** | **Clinical recovery** | **Personal recovery** | **Service-defined recovery** |
| --- | --- | --- | --- | --- | --- | --- | --- | --- |
| 1 | ASTON2012 | UK | In-patient | Nurses | 5 | X |  |  |
| 2 | GILBURT2013 | UK | Community and in-patient | Team leaders | 16 | X | X | X |
| 3 | TICKLE2012 | UK | Community and in-patient | Clinical psychologists | 11 | X | X | X |
| 4 | TURTON2010 | Europe | In-patient | MDT | 447 | X | X |  |
| 5 | FELTON2006 | USA | Community | Multidisciplinary | 212 | X | X | X |
| 6 | SULLIVAN2013 | USA | Community | Case managers | 40 | X | X |  |
| 7 | SULLIVAN2012 | USA | Community | Case managers | 40 |  | X |  |
| 8 | RICE2009 | USA | Community | Case managers | 11 |  |  | X |
| 9 | WATSON2011 | USA | Community and in-patient | Multidisciplinary | 109 | X | X | X |
| 10 | ROGERS2007 | USA | Community and in-patient | Psychiatrists | 24 | X | X | X |
| 11 | DUNLAP2009 | USA | Not specified | Social workers | 8 | X | X | X |
| 12 | COURTNEY2013 | Australia | Community | Social workers | 10 | X | X | X |
| 13 | VANLITH2009 | Australia | Community | Art therapists | 3 |  | X |  |
| 14 | CLEARY2013 | Australia | In-patient | Nurses | 21 | X | X | X |
| 15 | HUNGERFORD2013 | Australia | Not specified | Multidisciplinary | 18 |  | X |  |
| 16 | BATTERSBY2012 | Canada | Community | Multidisciplinary | 44 | X | X |  |
| 17 | SCHWARTZ2013 | Canada | Community | Multidisciplinary | 10 | X | X |  |
| 18 | KIDD2014 | Canada | In-patient | Multidisciplinary | 20 | X | X |  |
| 19 | PIAT2012 | Canada | Community and in-patient | Multidisciplinary | 68 | X | X | X |
| 20 | NG2008 | Hong Kong | Community and in-patient | Psychiatrists | 12 | X |  | X |
| 21 | KAEWPROM2011 | Thailand | In-patient | Nurses | 24 | X | X |  |
| 22 | CONE2012 | New Zealand | Community and in-patient | Occupational therapists | 10 |  | X | X |

**Online Data Supplement 2: Summary of included studies (n=22)**

| # | Database ID | Study ID | Country | Method | Participants/ Setting | Main Findings | Quality rating |
| --- | --- | --- | --- | --- | --- | --- | --- |
| 1 | 857 | ASTON2012 | UK | Focus groups were used to investigate what staff and service users say about the concept of recovery. The topic guide included questions like “what does the word recovery mean to you?” and “what do you think it would take for a recovery approach to work in local mental health services?” Inductive analysis was undertaken using Framework. | There were two focus groups; one with service users (N=6) and one with in-patient mental health nurses (N=5). Data from the service user and staff focus groups were kept separate during the analysis so that they could be compared. | Four central themes emerged:  1) understanding of recovery  The different meanings are described and a need for a shared understanding in order for recovery-orientated services to be delivered is argued.  2) semantics (the use of language to describe the processes of recovery)  Describes alternatives to the term ‘recovery’ suggested by participants.  3) therapeutics (relationships between nurses and patients)  Describes the difficulties nurses have with providing recovery support, such as not having enough time and being more familiar with task-oriented routines. Nurses also appeared to view recovery support as something they do to patients, rather than a collaborative process.  4) [recovery as] a journey.  Finally, both groups viewed recovery as “a journey”, not something that it is a quick fix but something that is a long and winding road. | 6/18 Low quality  Conclusions are not grounded in the data. Reflexivity, and auditability not addressed. |
| 2 | 6873 | GILBURT2013 | UK | Pre-post quasi-experimental (mixed methods) design. Within the study, semi-structured interviews were conducted with a sample of the training intervention group to assess their understanding of recovery and its implementation. Transcripts were analysed with inductive coding. | Semi-structured interviews were carried out with 16 team leaders in the intervention teams. The participating service providers were all working in community-based and in-patient rehabilitation adult mental health teams in the London boroughs of Lambeth, Southwark and Lewisham. | Nine themes emerged from the interviews; five related to the perception and provision of recovery-oriented care (care provision, the role of hope, the language of recovery, ownership and the need for multi-disciplinarity) and four related to its implementation (hierarchy, training, measures of recovery and resources). The researchers note that interviewees’ accounts often reflected a struggle to define recovery. | 9/18 = Mid quality  Detailed sample of interview participants not provided.  Research limitations regarding qualitative component not addressed.  Reflexivity, ethics and auditability not addressed. |
| 3 | Expert | TICKLE2012 | UK | Grounded theory study using semi-structured interviews to explore the views of clinical psychologists towards the concepts of “risk” and “recovery.” Three broad questions led the interviews: views on what constitutes risk, views on what constitutes recovery and how the concepts of risk and recovery might relate to each other. The analysis was inductive, beginning with codes that were close to the data. Codes became more focused as they were synthesised across transcripts and put into descriptive categories. Finally, categories were raised to theoretical concepts to describe relationships between categories. | 11 clinical psychologists working in adult MHS within two NHS trusts were recruited. Five worked within community mental health teams, two in specialist psychological services, one in rehabilitation services, one in acute mental health services, and two across multiple mental health services. | The four overarching descriptive categories were  1) Influences (resources, service models and beliefs about what is helpful for service users)  2) stakeholders  3) working with risk 4) the meaning of recovery.  The three theoretical categories were  a) Changing cultures in mental health services (e.g. emergence of recovery, increased accountability and blame, move away from paternalistic approaches)  b) dominant and marginalised concerns (e.g. risk of harm, fear of blame vs. benefits of recovery, learning from incidents)  c) professional conflicts and dilemmas (e.g. wanting to support service users vs. wanting to promote independence or wanting to increase responsibility of service user vs. awareness of professional accountability). The authors conclude that the clinical psychologists studied are aware of the emergence of recovery-orientated approaches but feel unable to incorporate them in practice because of perceptions of being bound by both their own limitations and those of their circumstances including issue of risk, thus giving rise to dilemmas in professional practice. | 11/18 = Mid quality  Sample design not reported.  Detailed analysis including exploration of diverse and negative cases. |
| 4 | 7634 | TURTON2010 | EUROPE | Three round Delphi consultation. 1) Participants were asked to list 10 items which “most helps recovery for people with long-term mental health problems in institutional care” 2) Respondents had to rate the items generated in the first round  3) Respondents had to rate the items again in light of feedback from the way the items were generated in round 2. Items with high ratings and within-group consensus were grouped into themed domains by the researchers. Domains with at least one item rated as essential with 100% group consensus were compared across stake holder groups and countries. | Four separate expert groups (staff, advocates, service users and carers) across 10 different European countries took part. Participants were known to have a recovery-orientation in institutional settings. Data collection and analysis between stakeholders was kept separate. | A total of 4,098 items were generated, of which 3,178 were rated as essential with at least 80% within group consensus. The items were group into 11 broad domains:  1) social policy, human rights and advocacy  2) social inclusion  3) self-management and autonomy  4) therapeutic interventions  5) governance  6) staffing  7) staff attitudes  8) institutional environment  9) meeting needs after discharge  10) involvement of caregivers  11) physical health care.  Therapeutic interventions had the most consensus across the countries amongst staff, which suggests a more clinical model of recovery (this domain scored high across all stakeholder groups). There were few differences between the stakeholder groups, although staff put a low emphasis on factors related to staffing issues and post discharge support compared to other groups. | 12/18 = Mid quality  Quality of method not fully captured by the qualitative appraisal checklist.  Diverse sample – although aim to achieve consensus.  Aimed to keep original wording of items close to participants’ language.  Discussion of weaknesses of some of the items. |
| 5 | 4833 | FELTON2006 | USA | Process evaluation of recovery principles and practices training. Two researchers observed the training sessions and took notes. Any input from a trainee (ACT staff) that directly challenged or endorsed a recovery-based principle/practice, that reflected a need for more information, or that was an example of a trainee’s use of recovery-orientated practice were coded to identify broad categories. The inputs were then categorised into the different categories. The researchers also noted whether the inputs were an example of the staff member’s work, comments about a client or simply reflected a more general experience. They also noted whether the input affirmed the value of recovery, suggested a struggle with working within recovery, or objected recovery or its applicability. Finally, they noted whether the input was made during introductory sessions or in follow-up sessions when it was hoped that trainees would have implemented wellness management or some recovery principles in their practice. | A total of 212 multi-disciplinary staff from 18 different agencies attended at least one training session. There was a total of 99.5 hours of training which took place and was observed between June and December 2004. | 205 trainee inputs were identified during the training sessions. Inputs fell into one of ten categories describing endorsement of or difficulties with recovery-orientated practice  1) Who is eligible for wellness management?  2) My clients won’t admit to having a mental illness  3) Crises prevent us from using recovery  4) Recovery means working with client-centred goals  5) Developing the recipient’s goals (reflects the need but also the challenges of working with goals) 6) Whose goals? (use of system-derived goals rather than client-centred goals).  7) Making a good connection  8) Who should do what?  9) Symptom dominant vs. holistic views of the clients, and  10) wellness works (reports of using the tools presented in the wellness management training program).  A majority of the trainees’ comments fit positively with recovery. The most positive inputs were those in describing service users in holistic terms and using techniques to achieve client-centred goals. Challenges were around establishing collaborative relationships with clients, especially those who deny having a mental illness. A subset of trainees did not seem able to abandon their notions about the primacy of medication and ADL improvement. Similarly, other trainees held the view that some of their clients were too sick for recovery. | 13/18 = High quality  Good coverage - two researchers attended the sessions and after attending 26 hours of meetings together they checked the accuracy of each other’s notes and refined their note-taking procedures.  Used constant comparison techniques in analysis.  Two researchers independently coded the transcripts from two of the sessions into the ten categories. Researchers achieved 80% agreement after the first transcript and this moved up to 87% with the second transcript. The validity of the ten categories was checked against data from two days of training in another state.  Explored perspectives along recovery orientation continuum. |
| 6 | 1578 | SULLIVAN2013 | USA | Semi-structured interviews were carried out with case managers. Each respondent was asked to describe the characteristics of the clients they served, aspect of the job they liked/disliked and their beliefs about the concept, process and possibility of recovery from mental illness. Data was analysed in parallel such that as interesting themes arose, these could be further explored in subsequent interviews. Based on the respondent’s answer to “Do you think the clients on your caseload can recover” transcripts were assigned to one of four levels of hopefulness (low, moderate, hopeful and high). Differences in the understandings of recovery and reported recovery-promoting behaviours were examined across these different levels of hopefulness. | 50 case managers working with adults with SMI were recruited from nine urban and suburban community mental health teams. Only 40 interviews were included in the analysis, either because of technical difficulties or because it transpired that the case managers worked with children. | 1) Low hope (N=6) case managers, who expressed little hope that recovery from mental illness was possible tended to view recovery as synonymous with “cure”. These case managers emphasised their role in helping to stabilise clients.  2) Moderate hope (N=12) case managers viewed recovery as gains in symptom management and social functioning and talked about the importance of patient insight and treatment compliance for recovery.  3) Hopeful case managers’ (N=8) definition of recovery mirrored current perspectives of the concept and was viewed as individually determined. Compared to the low hope group, control of the case management process has shifted from the professional to the consumer, who in turn is seen as capable of taking an active part in decisions that are critical to their well-being.  4) The high hope group (N= 14), consistently defined recovery as the ability of consumers to accomplish personal goals, and when describing their work emphasised the importance of a person-centred philosophy. | 12/18 = Mid quality  Convenience sample in restricted geographical area.  Carried out 9 interviews with ineligible participants!  Addressed inter-rater reliability by monitoring congruence between stated hope in recovery and ‘recovery’ practice examples.  Offers new classification of clinicians re: hope and how levels of hope affect practice. |
| 7 | 1808 | SULLIVAN2012 | USA | Semi-structured interviews were carried out with case managers. Each respondent was asked to describe the characteristics of the clients they served, aspects of the job they liked/disliked and their beliefs about the concept, process and possibility of recovery from mental illness. Data was analysed in parallel such that as interesting themes arose, these could be further explored in subsequent interviews. The focus of the analysis was on the process of helping, specifically the use of the professional relationship and other key elements of direct managers identified as relevant to working with this clientele. Each interview was coded by two reviewers using deductive framework initially and then using inductive coding. | 50 case managers working primarily with adults with serious mental illness. They were recruited from nine urban and suburban community mental health teams across two states. Only 40 interviews were included in the analysis, either because of technical difficulties or because it transpired that the case managers worked with children. | The main themes which characterise the helping relationship from the staff perspective were  1) engagement (listening to clients, seeing them as a person not a diagnosis, using the principles of client-centred planning and self-determination)  2) pushing, pulling and letting go (striking the right balance between helping the client without being too controlling and how this dynamic changes over the course of the relationship),  3) moving forward (instilling hope, talking about goals and recovery)  4) building on the relationship (being a stable presence in a client’s life whilst maintaining boundaries and not becoming too enmeshed). The authors argue that case management requires complex and nuanced professional skills. | 11/18 = Mid quality  Study aims not clear.  Large sample. No information on sampling strategy.  No discussion of ethical issues. |
| 8 | 2814 | RICE2009 | USA | Interviews and an interpretive phenomenological approach was used to understand the experiences of cases managers in delivering care to women diagnosed with schizophrenia. | 11 case managers were recruited from a community mental health centre that serves people diagnosed with severe mental illness. | The main themes which emerged from the data were that  1) case managers viewed themselves as being supportive  2) they felt overwhelmed and frustrated with their jobs, particularly they struggled to apply the tenets of recovery and understand their role in the process for women who have experienced violence and poverty in their lives. | 4/18 = Low quality  Very little information provided on analysis strategy.  Limited scope for drawing wider inference.  Research and sample design not defended. Quality of data collection and analysis not reported. |
| 9 | 1152 | WATSON2011 | USA | Ethnography using semi-structured interviews to examine meanings of recovery in New Mexico in light of a major state-wide reform of mental health services. The interview schedule included prompts for providers to share their thoughts and attitudes about official calls for recovery-orientated services. The interviews were coded using a framework based on the specific questions and broader domains that made up the interviews. | 109 interviews were carried with frontline adult mental health service providers in 14 community-based agencies across 6 counties in New Mexico. Six of the agencies were community mental health centres, three were substance abuse treatment centres, two were specialist outpatient services for homeless adults with co-occurring disorders and three were small group practices. The interviews took place between April and December 2006. | The major themes discussed were:  1) lack of clarity about the meaning and implementation recovery,  2) conflation of recovery with full symptomatic remission and scepticism that the latter is possible,  3) recovery as a rhetorical device that draws attention away from necessary infrastructure changes and driven by profit motives that underlie privatised managed care systems,  4) personal experience as a form of expertise in clinical settings and 5) stigma and discrimination as barriers to recovery. The authors argue that the term recovery serves as a symbol with many meanings, which can change depending on the desired agenda. | 8/18 Mid quality  Large sample using purposive sampling.  Identified capabilities approach - but unclear how this came from analysis – bit of a leap. Reflexivity, ethics and auditability not addressed. |
| 10 | 5078 | ROGERS2007 | USA | Three work-groups were formed at a conference to discuss practice issues in recovery. The main topics addressed by the groups were their barriers experienced in promoting recovery and recommendations for change. The barriers and recommendations were documented and discussed in a final meeting attended by all participants. Thematic analysis was used to classify the barriers into categories. | The conference took place over one day in January 2006 in Philadelphia and was attended by 24 psychiatrists from institutional and community settings who were clinicians, administrators, and educators. | Twelve barriers were identified which fell into three categories  1) psychiatry knowledge, roles and training  2) transforming public health systems  3) environmental barriers to opportunity. Participants made 22 recommendations to address these barriers through changes in policies, programs, psychiatric knowledge and practices. | 2/18 = Low quality  Research design is not defensible - one day symposium/discussion.  Credibility of findings is questionable.  Study is poorly reported (brief report) – no mention of sample design, sample composition, quality of data collection and analysis. Reflexivity, ethics and auditability not addressed. |
| 11 | 7704 | DUNLAP2009Thesis | USA | Used phenomenology to uncover the lived experience of social workers as they created and implemented discharge plans within the recovery perspective. In-depth interviews captured the social workers' experiences regarding the discharge process, with an emphasis on stigma, resources, and the integration of the recovery process. Each interview lasted between two and three hours. A retrospective review was a second method of data collection whereby participants completed an anonymous retrospective review of the last 10 discharged clients (data collected within one week of the interview). Questions included date of first discharge discussion, whether stigma was addressed, whether the client agreed with the plan, and self-rated outcome of the discharge plan. | 8 Social workers were selected through a convenience sample using a snowball technique. The selected sample was a purposeful sample. The inclusion criteria specified that participants were a licensed social worker with one year of experience post graduation. In addition, to ensure a recovery perspective, the participants must have received training on the recovery perspective. The eight participants consisted of six females and two males. Seven participants were white and one participant was of Asian descent. Of the social workers included in the study, the years of experience varied from 5 years to 35 years. | The analysis describes the unique lived experience of participants creating and implementing the discharge plan. The analysis found two essential structures named  1. Competing priorities  2. Conflict.  Four key components within the essential structures were identified  a. sharing and creating power  b. experiencing loss  c. feeling competent  d. handling conflict. The analysis of the key components identified that participants described competing dual responsibilities in the discharge planning process. To reduce role conflict, participants expanded their definition of discharge planning success to include the completion of the treatment goals as well as linkages to external supports. | 14/18 = High quality  Space to report study in full – dissertation thesis. Phenomenological inquiry.  Negative case sampling evident.  Ethical considerations not reported. |
| 12 | 1321 | COURTNEY2013 | AUSTRALIA | In-depth, semi-structured interviews were conducted to explore how social workers manage the tensions of working within a recovery-orientated approach with involuntary clients on Community Treatment Orders. The interview schedule asked participants to describe their practice approaches and the principles that guide their work with involuntary clients, their understanding of recovery, how they manage their work with involuntary clients including how they manage and overcome any challenges. Thematic analysis was used to analyse the interviews. Whilst the analysis was primarily inductive, a framework of recovery principles (Davidson et al., 2005; the development of new meaning and purpose, valuing lived experience, overcoming disability, developing relationships, challenging discrimination, the development of agency and empowerment) was later applied to the data in order to determine which recovery principles the social workers were drawing on. | Interviews were carried out with 10 social workers recruited from a variety of community mental health teams in Adelaide. Participants had varying levels of work experience in mental health ranging from 1-15 years. | Three core themes were identified  1) embedding involuntary treatment within a recovery approach (e.g. communicating in a non-authoritarian way, building a positive relationship)  2) professional resistance and managerial agendas (attitudes of non-social work colleagues, the dominance of the biomedical model in multi-disciplinary teams, the view that recovery is used as a means of removing responsibility from themselves and putting it onto the clients)  3) challenging resistance and enhancing self-determination (building an element of choice into CTOs) | 11/18 = mid quality  Position research within an epistemology (social constructionism), but no evidence of theoretical position influencing study design, analysis etc.  Some discussion of social work values and recovery values – assuming authors are social workers. |
| 13 | 263 | VANLITH2009 | AUSTRALIA | Phenomenological open-ended interviews were, conducted with art facilitators to elicit their conceptualisations, experiences and reflections of the contribution of art making to recovery. The data was analysed using IPA. | Three facilitators were recruited from different community-based art making programs in Australia. | Eight themes and three domains emerged from the data. The first domain was the skills, qualities and approaches of the facilitator seen to help recovery which included  a) active witnessing and beliefs in the client’s creative emergence,  b) creating learning opportunities, and c) providing a space to experiment. The second domain identified the ways in which art making can be a transformative experience for clients which includes  a) transcendence and satisfaction  b) self-reflection  c) giving form to feeling. Finally, the third domain related to how art making assisted the individual to connect with the world by  a) generating passion and meaning  b) creating relationships with others. | 11/18 = Mid quality  Described as a pilot study  Detailed profile of sample  Evidence of IPA use – context retained in analysis.  Small sample.  Reflexivity, ethics and auditability not reported. |
| 14 | 2 | CLEARY2013 | AUSTRALIA | Interviews were carried out with acute inpatient mental health nurses to explore their understanding of recovery and how they incorporate the recovery paradigm into practice. The interview guide was developed following a literature review and discussions with the research team. Responses were analysed using thematic analysis. | 21 nurses working in one of four acute inpatient mental health services were recruited. Data collection continued to theoretical saturation. | Three main themes emerged:  1) perception of recovery  The most prominent perception of recovery was that of holism where social factors, psychological factors and living skills were viewed as important to recovery. Other perceptions of recovery were also discussed.  2) humanism  When asked about the most important contribution nurses make to recovery, responses primarily clustered around the notion of humanistic interpersonal nursing such as developing therapeutic relationships, being kind, discussing options etc  3) practical realities Nurses reported that they support recovery (in order of frequency) using medication, education, goals/discharge planning, interpersonal relationships, and social and practical aspects of daily living. | 7/18 Mid quality  Convenience sample used.  Two members of the research team analysed the data. No mention of data management method. Limited analysis presented without discussion of how concepts were constructed. Categories not illustrated with quotes. |
| 15 | Expert | HUNGERFORD2013 | AUSTRALIA | Single-case embedded study. Focus groups were conducted to assess staff experiences of the implementation of recovery support. One semi-structured interview was carried out with a participant who did not feel they would be able to respond honestly or openly in front of other group members. The focus group/interview schedule was the same across groups and asked participants to discuss their experience of the implementation of recovery support; the benefits and challenges of this process; and their ideas on how the process might have been or could be improved. Each of the transcripts were analysed independently to identify the emerging themes, which were then refined and comparatively analysed across the groups. | Participants were recruited from a single public mental health service in Australia in 2010. Four focus groups were conducted for each; nurses, occupational therapists, social workers and psychologists (approx. 3 participants per group). A separate focus group for managers was conducted including both area-wide and front-line managers (N=5). It is unclear how many people participated in total. | Two major themes were identified; “change management” and “work practices”.  “Change management” describes the tension between practitioners and managers in the implementation of recovery support. Many practitioners felt frustrated at the top-down approach taken by managers and their failure to acknowledge that recovery is not a new concept for them. Meanwhile managers believe that practitioners are pessimistic and their past practices less than satisfactory. “Work practices” describes the view held by practitioners that a lack of community-resources, under-staffing and too much paperwork challenge the implementation of recovery. In contrast, managers viewed these concerns as symptoms of anxiety amongst practitioners about the changes occurring. | 10/18 = Mid quality  Detailed profile of achieved sample not reported.  The interviews were analysed independently by two researchers who then compared their results (unclear how any disagreements were resolved). |
| 16 | 2016 | BATTERSBY2012 | CANADA | This study is a feminist- informed ethnography. Methods included conducting interviews and focus groups with staff and service users. The aim of the research was to produce descriptions of what happened to people during the process of being transferred from a large institution which was closing down to recovery-orientated tertiary and community-based facilities. Data from the ethnography were organised and coded into emergent themes. Gender-based and intersectional analytic frameworks were also applied to pose questions about the similarities or differences among the needs of women and men. | 44 individual interviews and 3 focus groups were carried out with mental health managers and staff working in tertiary and community-care facilities in one of two towns. | Individuals who had been involved in the initial planning of the new facilities described how they had hoped to implement psychosocial rehabilitation within the context of a recovery philosophy that created space for maximum patient autonomy and decision making. However, they also reported that this would be a challenge given the level of need amongst people who have been long-term institutionalised. There was evidence to suggest that the staff found it difficult to move away from a more custodial model of care in practice. This was also true for service managers who struggled with letting service users make decisions while assessing their own risk management and legal responsibilities. Interviews with staff reflected inconsistencies in the understanding of recovery. For example, staff believed that PSR did not apply to service users who were too ill (which conflicts with the values of PSR and recovery). | 14/18 = High quality  Large diverse sample.  Part of a wider ethnographic study.  Clear theoretical underpinning.  Report combines findings and discussion sections. Weak conclusion. |
| 17 | 6954 | SCHWARTZ2013 | CANADA | In line with the principles of participatory action research, the participants agreed on the research design together and decided to use narrative phenomenology. Participants met up for weekly discussion groups for 10 weeks and generated stories about recovery from provider and consumer perspectives. They dialogued about the values underlying the stories to understand what really matters in recovery. The sessions were transcribed and analysed for themes. | The sample consisted of 10 stakeholders (3 consumers, 2 qualified peer support workers, 3 occupational therapists, 1 psychiatrist and 1 clinician- researcher. In line with participatory action research the sample consisted of the participants who had raised the initial research question from an outpatient mental health clinic at a university hospital. | The central themes were  a) relational space (boundaries, relationships, limited resources, shifting roles, power)  b) obstacles (stigma, culture, fear of exposure); c) meaning making (what is recovery?). Providers frequently voiced stories about the intrapersonal conflict between the “need to protect and the desire to support consumer autonomy. Another tension underlying provider stories was between the desire for connection with consumers and the need to “maintain professional boundaries.” | 7/18 = Mid quality  Brief report of preliminary findings.  Provide a summary of main themes without quote illustrations.  No attention to reflexivity, ethics or auditability. |
| 18 | Web of Science Citation search | KIDD2014 | CANADA | Pre- post- test experimental design using mixed methods. The intervention was a series of talks to staff by 12 former patients. At post-test focus groups were carried out with the participants in the intervention group about their experiences with the speaker series and the data was analysed using content analysis. | 20 staff working in one of 6 inpatient units in a large urban psychiatric facility in Canada. The units were matched as closely as possible according to average length of stay and one unit from each pair randomly allocated to either the intervention (series of talks) or the control group. | Staff who had attended the talks showed a significant increase in recovery knowledge, which was not evident in the control group. This is thought to reflect a greater understanding that recovery is nonlinear and can includes a number of different pathways and resources. Two themes emerged from the qualitative data: 1) the talks gave staff hope, both for clients and for their role and 2) they spurred staff to reflect on their practice. Overall, staff had a greater appreciation for the potential of inpatient clients and a greater recognition of the importance of engaging clients at a human level by recognizing their individuality and treating them with respect. | 8/18 = Mid quality  Qualitative component of mixed methods study reported in brief.  Limited quotes. Unclear how conclusion is derived from data. |
| 19 | 1946 | PIAT2012 | CANADA | Focus groups were conducted and participants were asked a series of open-ended questions: How do you define recovery? How do the services provided by your team reflect recovery-orientated practice? And, how is recovery-orientated practice part of your day to day work? A socio-demographic questionnaire was also administered at the start of each group. The data was first coded according to two broad themes: 1) reacting and responding to recovery-orientated practice, and 2) implementing recovery orientated practice. The data was then coded inductively using an open coding process and themes were identified. | The nine focus groups were conducted with a sample of 68 service providers recruited from three Canadian sites. At the time of the study, all three sites were in the early stages of implementing recovery-orientated practice into their organisation. The participants were staff providing direct service to people with serious mental illness (e.g. social workers, psychologists, occupational therapists) at various levels of service delivery (e.g. frontline, clinical supervisors, team leaders, managers) and working in diverse settings (inpatient and outpatient). | Three major themes were identified  1) Service providers had positive attitudes towards recovery-orientated reform (e.g. a better way of delivering services, represent a change in the power relationships focus)  2) some expressed scepticism (e.g. just another buzzword, doesn’t contribute anything new to practice).  3) challenges associated with the implementation of recovery (e.g. the conceptual uncertainty and consistency regarding the meaning of recovery, limited organizational support and leadership, the applicability of recovery to certain populations, bureaucratic burdens of recovery and stigma/social exclusion of people with mental illness). | 14/18 = High quality  Sample included a range of stakeholders/levels of delivery.  The data analysis was carried out by the team and involved constant team discussion to ensure agreement. |
| 20 | Hand search of included references | NG2008 | HONG KONG | Focus groups were conducted to examine trainee psychiatrist views of recovery from schizophrenia. The interview schedule was developed by a team of mental health professionals and included questions such as “How do you define recovery from schizophrenia?”, “Do you think patients suffering from schizophrenia can fully recover?” and “What can others do in promoting their recovery?”. Transcripts were analysed using content analysis. | Two focus groups were conducted with 12 trainee psychiatrists working in various psychiatric units in Hong-Kong. One group contained newly trained psychiatrists with less than two years clinical experience (n=6) and the other with more experienced psychiatrists who had five or six years of experience (n=6). | Four themes emerged  1) Absence of relapse is a pre-requisite for recovery  2) recovery means different things to different people at different stages of illness  3) Recovery is an important agenda item (but for most participants this meant discussing medication use and risk of relapse).  4) recovery in the presence of persistent symptoms (this was more often endorsed by junior psychiatrists). The central theme encompassing the four categories was that recovery is a process that is complex and difficult to define. | 9/18 = mid quality  Data management method not reported. Ethics e.g. consent procedures not mentioned. Some reporting of limitations of study. |
| 21 | 863 | KAEWPROM2011 | THAILAND | Semi-structured interviews were conducted in which nurses were asked to share their opinions about recovery from schizophrenia, as well as the factors that are involved in recovery. The data was analysed using thematic analysis. | 24 mental health nurses were recruited from two general hospitals and one psychiatric hospital in Thailand. For inclusion, nurses needed to have been providing care for at least one year to people with a diagnosis of schizophrenia. The nurses’ experience ranged from 3-27 years. | Views of recovery support were characterised by a focus on clinical and functional improvement, notably symptom remission, an ability to carry out activities of daily living and a return to work or study. Four themes described the factors thought to be involved in the recovery process  1) personal facilitators (hope, acceptance, and treatment adherence)  2) environmental facilitators (family, a supportive community, good access to mental health services)  3) personal barriers (poor adherence to medication, illness-related factors)  4) environmental barriers (stigma, lack of integration of mental health services). | 7/18 = mid quality  Purposive sampling.  Analysis weak – no attention to negative cases.  Conclude that nurse understanding is dominated by clinical recovery examples – unsure that this accounts for all the data. |
| 22 | 9399 | CONE2012 | NEW ZEALAND | Focus groups and interviews were conducted to explore how occupational therapists incorporate the recovery approach into mental health practice. The interview guide included: how do you incorporate the recovery approach into your practice? What facilitated and what challenged the incorporation of the recovery approach into practice? How do you use occupations to facilitate recovery, to empower people and inspire hope? How is the recovery approach useful and/or not useful in the New Zealand context? Thematic analysis was used to code the data. | 10 occupational therapists working in New Zealand were recruited, and allocated to one of two focus groups to ensure that a diversity of work experience and settings were represented. Three participants were then purposely selected from the focus group for in-depth follow up interviews based on their professional experience and contributions to the emerging main themes. | The main themes discussed were incorporating the recovery approach into practice, philosophical congruence supports recovery orientated practice and challenges with incorporating recovery into practice. Overall, the occupational therapists incorporated recovery into their practice in a variety of ways, notably through the process of facilitating occupational engagement on the ward, in the community and on an ongoing basis in life. They perceived the recovery approach to be an integral part of occupational therapy practice in mental health, but acknowledged that this could be challenging. | 6/18 = Low quality  Sampling method and details of settings, and participant characteristics not reported.  Weak analysis – Diversity and multiple perspectives not described.  Study limitations addressed e.g. gaps in sample. |

**Online Data Supplement 3: Data Extraction Table (n=22)**
